# Symptom Trajectories and Long-Term Sequelae of COVID-19: A Matched Case–Control Study with Population-Based Controls

**DOI:** 10.3390/jcm15103707

**Published:** 2026-05-12

**Authors:** Sebastian Sołomacha, Maciej Alimowski, Anna Moniuszko-Malinowska, Łukasz Kiszkiel, Piotr Laskowski, Marlena Dubatówka, Paweł Sowa, Karol Kamiński

**Affiliations:** 1 Department of Population Medicine and Lifestyle Diseases Prevention, Medical University of Bialystok, Waszyngtona 15B, 15-269 Bialystok, Poland; sebastian.solomacha@gmail.com (S.S.); marlena.dubatowka@umb.edu.pl (M.D.); pawel.sowa@umb.edu.pl (P.S.); 2 Society and Cognition Unit, University of Bialystok, Plac NZS 1, 15-403 Bialystok, Poland; m.alimowski@uwb.edu.pl (M.A.); l.kiszkiel@uwb.edu.pl (Ł.K.); p.laskowski@uwb.edu.pl (P.L.); 3 Department of Infectious Diseases and Neuroinfectious, Medical University of Bialystok, 15-089 Bialystok, Poland; anna.moniuszko-malinowska@umb.edu.pl; 4 Department of Cardiology and Internal Diseases, Medical University of Bialystok, ul. Sklodowskiej 24a, 15-276 Bialystok, Poland

**Keywords:** SARS-CoV-2, COVID-19, post-COVID-19, long COVID-19, symptom trajectories

## Abstract

**Background/Objectives:** Post-COVID-19 condition involves heterogeneous, multisystem symptoms with uncertain recovery. We characterized symptom trajectories from hospitalization to approximately 4 weeks and to 6–8 months and compared the 6–8-month symptom burden with season-matched controls, accounting for serology-identified, previously unrecognized infections. **Methods:** An individually pair-matched case–control study of adults with RT-PCR–confirmed SARS-CoV-2 and population controls from the Bialystok PLUS cohort, matched on age, sex and a two-month visit window, was performed. All participants underwent anti-nucleocapsid serology. Hospitalized cases were reassessed at approximately 4 weeks and 6–8 months. Cross-sectional outcomes used non-parametric tests and multivariable regression; longitudinal change used paired tests and generalized estimating equations. **Results:** We included 402 adults (201 post-COVID-19; 201 controls). In hospitalized cases, respiratory symptoms declined rapidly by approximately 4 weeks and remained low at 6–8 months; smell/taste recovered more slowly; fatigue improved modestly; anxiety changed minimally. At 6–8 months, total symptom counts were higher in post-COVID-19 than in controls (median 4 vs. 2), with serology-positive controls intermediate (median 3). Excess burden was concentrated in non-respiratory domains (fatigue, neurocognitive, cardiovascular, and dermatologic), whereas respiratory differences were not significant. In the multivariable model, female sex remained an independent predictor of higher multisystem burden, whereas age, body mass index, hospitalization, and acute biomarker severity were not associated. **Conclusions:** Six to eight months after symptomatic COVID-19, multisystem symptom burden remains substantial relative to season-matched controls, despite substantial resolution of respiratory complaints. Serology-based identification of previously unrecognized infections indicates an intermediate burden and can guide targeted follow-up.

## 1. Introduction

Since its emergence, infection with severe acute respiratory syndrome coronavirus 2 (SARS-CoV-2) and the resultant coronavirus disease 2019 (COVID-19) have posed an unprecedented challenge to healthcare systems worldwide. Researchers and clinicians initially focused their efforts on the acute phase of the infection and on treating life-threatening complications, including severe respiratory failure and acute respiratory distress syndrome (ARDS). Since then, however, it has become clear that a substantial proportion of patients experience persistent symptoms affecting multiple body systems, which may endure for months to years after the initial illness. This condition is referred to as post-COVID-19 condition (PCC), commonly known as long COVID [1,2].

The prevalence of PCC varies considerably depending on the study design, population, and duration of follow-up. In a systematic review of more than 50 studies, Lopez-Leon et al. identified more than 50 long-term sequelae, the most common of which were fatigue (58%), headaches (44%), and attention deficit disorder (27%) [3]. Longitudinal studies have confirmed that these problems can persist well beyond the first year. Huang et al. demonstrated that at 6 months after hospital discharge, 63% of patients reported fatigue or muscle weakness. Meanwhile, 23% reported anxiety or depression [4]. A two-year follow-up of the same cohort showed that survivors of COVID-19 continued to experience mobility problems, pain, and reduced quality of life compared with the control group. Similarly, persistent dyspnea, fatigue, and impaired quality of life at 12 months after infection were documented by Evans et al. [5].

The range of long-term effects is wide and includes the respiratory, cardiovascular, neurological, and psychological systems [3,6]. Taquet et al. evaluated a large retrospective cohort of over 230,000 patients. It found that 33.6 percent of COVID-19 patients received a new neurological or psychiatric diagnosis within 6 months [7]. Systematic reviews highlight fatigue, dyspnea, cognitive impairment, sleep disturbances, and mood symptoms as the most frequently reported manifestations [3,6].

Several factors were proposed as predictors of PCC. Elevated inflammatory and coagulation markers such as C-reactive protein (CRP), interleukin-6 (IL-6), and D-dimer are strongly correlated with the severity of the acute disease [8,9], but their role in predicting long-term outcome is less clear [10]. Clinical risk factors, such as female gender, older age, and higher body mass index (BMI), have been associated with an increased risk in some cohorts [11], but the results from individual studies remain variable. Moreover, the burden of symptoms during the first week of infection has been found to be a strong predictor of long-term COVID-19 in community-based cohort studies [11].

A notable limitation of previous research was the lack of studies examining symptom trajectories across different stages, from acute infection to early post-acute and up to several months after treatment. Most available analyses focus on a single time point, typically 3 or 6 months [12,13], which may overstate the development of symptoms and preclude characterization of the dynamic symptom transitions that occur during the post-acute phase [14]. However, several longitudinal studies followed patients from the acute phase to 12 to 24 months after infection [15]. In these studies, it is emphasized that long-term symptoms of COVID-19 may persist from the onset of the disease or emerge months after infection [16]. Even among such cohorts, few have systematically captured the early post-discharge transition (~4 weeks) alongside both the acute and late post-acute phases—a window critical for distinguishing transient post-acute phenomena from genuinely persistent disease. Our study fills this gap by focusing on three key phases: acute, early post-infection period, and 6 to 8 months post-infection.

Another significant limitation of the available studies is the lack of consistently selected control groups. Many studies relied on non-matched controls or convenience samples, which limited the ability to separate COVID-19-specific effects from background population phenomena [17]. Seasonal changes in respiratory symptoms, such as cough, nasal congestion, or dyspnea, are well documented [18] and, if not adequately controlled, can complicate the interpretation of post-COVID-19 complaints. In a nationwide Scottish cohort, more than half of never-infected controls reported at least one putative post-COVID-19 symptom at six months, illustrating the magnitude of this background-symptom confounding [19]. In addition, little is known about asymptomatic SARS-CoV-2 infections: although the risk of complications appears to be lower in these individuals, the infection can be mild yet persistent [20]. Meta-analytic estimates indicate that approximately 17% of asymptomatically infected individuals continue to report at least one symptom in the long term [21], yet few comparative studies have applied systematic anti-nucleocapsid serology to reclassify their control population accordingly. The present study addresses these gaps by combining longitudinal symptom assessment with season-matched, serology-verified population controls.

The primary objective of this study was to characterize symptom persistence in individuals with confirmed COVID-19. In addition, we evaluated the effect of seasonality on delayed sequelae reporting by comparing post-COVID-19 participants with season-matched population controls. Finally, we examined the occurrence of late complications in control subjects who were seropositive but had unrecognized prior infection to determine whether the long-term effects were specific to COVID-19-susceptible individuals.

## 2. Materials and Methods

### 2.1. Study Design

The study was conducted in Bialystok, Poland, at the Medical University of Bialystok and within the population-based Bialystok PLUS cohort. The study population comprised two separate cohorts. The first group comprised individuals who had recovered from COVID-19, confirmed by RT-PCR (reverse transcription polymerase chain reaction) using nasopharyngeal swabs and the CFX96 Real-Time PCR Detection System with a C1000 thermal cycler (Bio-Rad Laboratories, Hercules, CA, USA). This group included hospitalized and non-hospitalized cases, with the acute infection phase occurring between April 2020 and February 2022. Among hospitalized patients, trained medical staff collected structured data on 15 clinical symptoms occurring during an acute episode. In addition, routine laboratory tests performed during hospitalization provided measurements of C-reactive protein (CRP), interleukin-6 (IL-6), and D-dimer levels, which were used to assess inflammation and blood clotting and to classify the severity of the acute phase. Approximately 4 weeks after hospital discharge, participants attended scheduled follow-up visits at the infectious disease clinic, where the early sequelae of the acute phase of the disease were systematically assessed and documented. In accordance with the study protocol, all patients were systematically invited for a follow-up visit 6 to 8 months after the acute episode, during which a structured medical history was taken, and a comprehensive diagnostic evaluation was performed. Laboratory tests included the quantification of IL-6 and CRP. IL-6 concentrations were measured using the Elecsys IL-6 immunoassay (electrochemiluminescence immunoassay, ECLIA) on a cobas e 411 analyzer (Roche Diagnostics, Rotkreuz, Switzerland). CRP concentrations were measured using a Roche CRP assay on a cobas c 111 analyzer (Roche Diagnostics, Rotkreuz, Switzerland) based on an immunoturbidimetric method.

The second cohort consisted of a control group recruited from the population-based study Bialystok PLUS, which was examined between January 2020 and December 2022. Participants in this group reported no prior history of COVID-19. Matching criteria included age and sex as well as the timing of study visits. Each participant in the post-COVID-19 group was matched with a participant from the control group who had been examined during the same two-month period. This approach was adopted to minimize potential bias arising from seasonal fluctuations in the incidence of respiratory and systemic symptoms, thereby strengthening the internal validity of the comparisons between groups. In accordance with the study protocol, all participants—both from the COVID-19 recovery cohort and the control cohort—underwent serological testing during study visits using the Elecsys Anti-SARS-CoV-2 assay on a cobas e immunoassay analyzer (Roche Diagnostics, Rotkreuz, Switzerland). This double-antigen sandwich ECLIA employs recombinant nucleocapsid (N) antigen and detects total antibodies, including IgG and IgM, indicative of prior SARS-CoV-2 infection. Based on serological results, 64 of 201 individuals in the control group were identified as seropositive despite denying prior COVID-19 infection, indicating previously unrecognized infection. Uniform exclusion criteria were applied across both groups and encompassed a history of myocardial infarction, stroke, psoriasis, vitiligo, type 1 diabetes, Crohn’s disease, allergic diseases, chronic obstructive pulmonary disease, HIV infection, hepatitis B or C, thyroid dysfunction, rheumatoid arthritis, systemic lupus erythematosus, or cancer. A complete tabulated summary of inclusion, matching, and exclusion criteria is provided in Supplementary Table S1.

**Figure 1:**
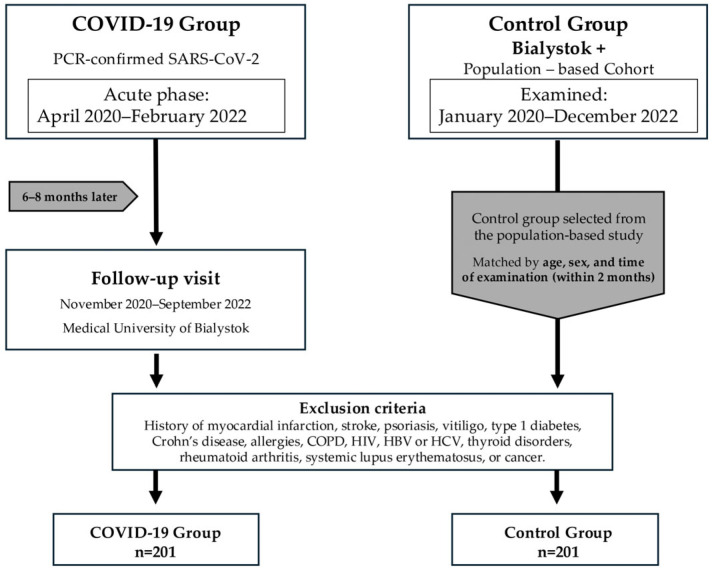
Study design and participant selection.

Following application of inclusion and exclusion criteria, the final study population consisted of 201 post-COVID-19 patients, of whom 161 had been hospitalized for SARS-CoV-2 infection, and 201 matched controls (Figure 1).

### 2.2. Symptom Assessment at 6–8 Months

At the 6–8-month follow-up visit, participants underwent a structured medical interview focusing on persistent or newly emerging symptoms following SARS-CoV-2 infection. The interview covered symptoms such as weakness/fatigue, cough, dyspnea, smell/taste disturbances, cardiac symptoms, thromboembolic complications, concentration problems, headache, memory impairment, blood pressure fluctuations, joint pain, anxiety symptoms, low mood/sadness, and hair loss.

For the purposes of this analysis, the findings regarding symptoms were defined primarily based on patient-reported symptoms recorded during a structured interview. At the time the study protocol was developed in early 2020, no condition-specific validated instrument for assessing post-COVID-19 symptoms was available; the Post-COVID-19 Functional Status scale [22] addresses functional status rather than symptom burden, and the first psychometrically validated symptom-specific scale (the COVID-19 Yorkshire Rehabilitation Scale) was not published until late 2021 [23]. We therefore developed a structured symptom inventory designed to comprehensively cover seven organ systems, administered by trained research personnel using a standardized script and applied identically to participants in the post-COVID-19 and control cohorts to minimize differential measurement bias. The seven symptom domains were defined a priori before statistical analysis and were based on clinical judgment rather than data-driven clustering. Individual symptom items were grouped into anatomically or functionally related domains to reflect major organ-system patterns of post-COVID-19 complaints and to improve interpretability of multisystem symptom burden. This approach also reduced the emphasis on isolated item-level comparisons, particularly for less frequent symptoms. The full mapping of individual symptom items to domains is provided in Supplementary Table S3. In addition, selected participants underwent further clinical tests as part of a comprehensive follow-up assessment, including olfactory testing (Sniffin’ Sticks test) for smell disturbances, body plethysmography for dyspnea, the Test Your Memory (TYM) for complaints regarding memory, as well as electrocardiography, blood pressure measurements, and cardiopulmonary exercise testing. However, the detailed quantitative results of these studies have not been included in these analyses and have not been used to reassess the status of the symptoms in this report. Findings from the psychophysical Sniffin’ Sticks olfactory testing in subsets of this cohort have been reported separately [24]. The present manuscript focuses, by design, on patient-reported symptom trajectories and burden.

### 2.3. Statistical Analysis

All statistical analyses were conducted using IBM SPSS Statistics version 26.0 (IBM Corp., Armonk, NY, USA) and Microsoft Excel version 16.108.1 (Microsoft Corp., Redmond, WA, USA). We applied 1:1 nearest-neighbor propensity score matching (without replacement) to pair post-COVID-19 cases with controls. Propensity scores (the probability of being a case) were estimated by logistic regression (including age and sex), with exact constraints on the two-month calendar window of the examination to mitigate seasonal confounding.

Longitudinal change in 5 prespecified symptoms in the hospitalized cohort (Hospitalization, 4 weeks, 6–8 months) was assessed within person using McNemar’s test for T2 vs. T1, T3 vs. T1, and T3 vs. T2 (two-sided exact binomial p; continuity correction +0.5 when b or c = 0), with multiplicity controlled by Holm across 15 tests. As a model-based complement, GEE with a logit link and an exchangeable working correlation estimated age- and sex-adjusted time effects (time categorical, T1 as reference), reported as aORs with 95% CIs.

For the 6–8-month composite outcome (23-item symptom count), between-group differences were tested with Mann–Whitney U (post-COVID-19 vs. control) and Kruskal–Wallis (three-group comparisons, e.g., anti-N subgroups), with pairwise Mann–Whitney post hoc tests and Holm correction; effect size: Cliff’s delta.

We compared baseline characteristics between post-COVID-19 and control groups using the Wilcoxon rank-sum test for continuous variables (reported as mean ± SD and median [IQR]) and χ^2^ (or Fisher’s exact when expected counts < 5) for categorical variables (n, %). For each symptom domain, we analyzed the prevalence of ≥1 symptom using multivariable logistic regression (exposure: post-COVID-19 vs. control; covariates: age, sex, BMI) and reported adjusted odds ratios (aORs, 95% CI; Wald two-sided p). Domain burden (proportion of positive items among items answered) was summarized as median [IQR] and compared with the Wilcoxon rank-sum test (Table S4). At the item level, we fitted similarly adjusted logistic models; within each domain, we controlled multiplicity using Benjamini–Hochberg FDR. Multiplicity was controlled within predefined families of analyses: Holm correction was used for longitudinal within-person comparisons and post hoc pairwise tests, while item-level analyses were adjusted using within-domain Benjamini–Hochberg FDR. Prespecified domain-level analyses were interpreted using adjusted estimates, 95% CIs, and consistency with item-level findings. Among post-COVID-19 participants, determinants of multisystem symptom burden (count of positive domains, 0–7) were evaluated with negative binomial regression, yielding incidence rate ratios (IRR, 95% CI). All tests were two-sided with α = 0.05; percentages used available cases, and models used complete-case analysis. Descriptive summaries were based on available data, with denominators reported where data were incomplete. For symptom-domain analyses, participants were included if data were available for the respective domain, and domain positivity was defined as at least one positive symptom item within that domain. Multivariable regression models were fitted using complete-case analysis for the variables included in each model. No multiple imputation was performed. Smoking-adjusted models were considered exploratory because smoking data were incomplete.

## 3. Results

### 3.1. Characteristics of the Study Group

We studied 402 participants (201 post-COVID-19; 201 controls). The groups were well balanced for age (median, 52.0 years in both; *p* = 0.416) and sex (female, 46.8% in each; *p* = 1.000). BMI was higher in the post-COVID-19 group (mean 29.8 ± 6.1, median 29.3) than in controls (mean 27.2 ± 5.4, median 26.6; *p* < 0.001). Current smoking, among participants with available questionnaire data, was less frequent in the post-COVID-19 group than in controls (21/171 [12.3%] vs. 53/158 [33.5%]; *p* < 0.001). The bimonthly calendar window of visit, used as the temporal matching criterion, was perfectly balanced between groups (*p* = 1.000), confirming that 1:1 matching successfully eliminated calendar-time imbalance. Anti-N seropositivity was expectedly higher post-COVID-19 (96.8%) than in controls (46.7%; *p* < 0.001). These positive results in the control group are consistent with previously unrecognized SARS-CoV-2 infections, despite the self-reported absence of prior COVID-19 (Table 1).

Among post-COVID-19 participants, 161/201 (80.1%) were hospitalized during the acute episode (Table 2). Using the composite biomarker definition (CRP ≥ 100 mg/L, IL-6 ≥ 80 pg/mL, D-dimer ≥ 1000 ng/mL), approximately one in six hospitalized participants met the criteria for severe COVID-19. Peaks of CRP, IL-6, and D-dimer showed substantial dispersion, with D-dimer values clustering near the threshold and upper quartiles of CRP/IL-6 exceeding it.

### 3.2. Longitudinal Symptom Trajectories

**Figure 2:**
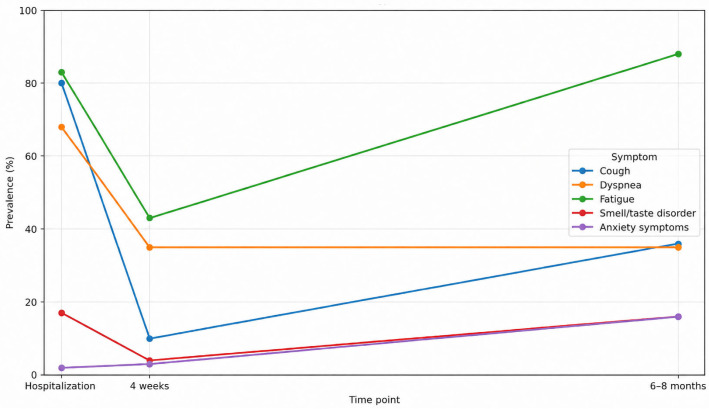
Longitudinal trajectories of symptom prevalence across hospitalization, 4 weeks, and 6–8 months (hospitalized cohort). Prevalence (%) with 95% confidence intervals for five symptoms—cough, dyspnea, fatigue, smell/taste disorder, and anxiety—assessed at three time points (hospitalization, 4 weeks, 6–8 months) in the hospitalized cohort. Estimates are based on binomial proportions with Wilson 95% CIs; inferential comparisons over time were evaluated using paired within-person analyses (McNemar) and age/sex-adjusted GEE models (logit).

In the hospitalized cohort, the temporal profiles of five prespecified symptoms—cough, dyspnea, fatigue, smell/taste disorder, and anxiety symptoms—showed distinct patterns across hospitalization, 4 weeks, and 6–8 months. As depicted in Figure 2, the overall burden shifted from an acute, predominantly respiratory presentation to fewer, more selective complaints at follow-up. Prevalence estimates are displayed with 95% CIs and annotated denominators (*n*/*N*) at each time point.

Cough declined most rapidly. From high frequency during hospitalization, prevalence dropped markedly by 4 weeks and remained low and stable at 6–8 months, with little evidence of late re-emergence. Dyspnea followed a similar trajectory: a pronounced early decrease from the acute phase to 4 weeks sustained at the final visit. These trends indicate that, for most patients, respiratory symptoms improved early, and the improvement persisted.

Smell/taste disorder demonstrated a steady, monotonic decrease across visits, yet a non-trivial subset continued to report disturbance at 6–8 months, consistent with slower or incomplete sensory recovery in a fraction of patients. Fatigue also decreased over time but less steeply than respiratory or sensory symptoms; despite improvement by 4 weeks, a residual burden remained at 6–8 months, suggesting protracted resolution. Anxiety symptoms changed least across assessments, with only modest variation and a generally flat trajectory over time.

These visual patterns are supported by within-person contrasts and age/sex-adjusted longitudinal estimates reported in Supplementary Table S2. Paired changes between adjacent time points confirm large, early improvements for cough and dyspnea that are maintained through 6–8 months; for smell/taste disorders, they show consistent movement toward recovery while quantifying the minority with persistent symptoms at the last visit. Adjusted time effects align with the unadjusted trajectories, showing a rapid and durable resolution of respiratory features, gradual but incomplete improvement in sensory function, slower decline of fatigue, and relative stability of anxiety across the follow-up window.

### 3.3. Six-Month Symptom Profile Across Groups: Prevalence, Distributions, and Determinants

**Figure 3:**
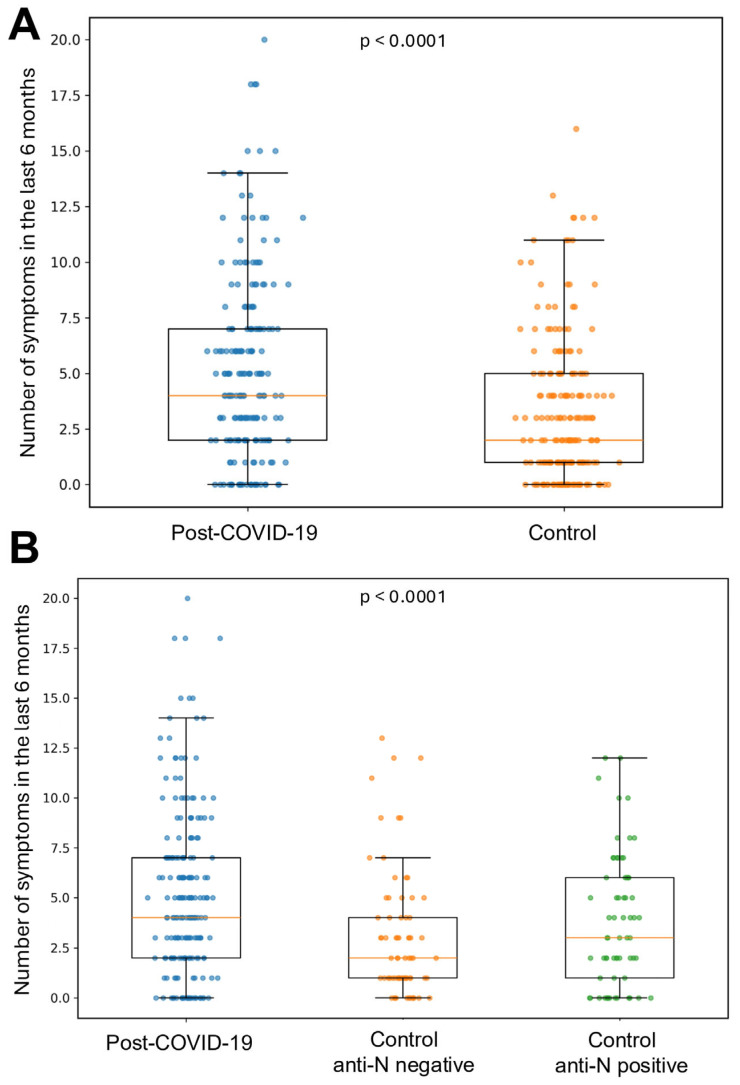
Number of symptoms in the last 6 months. (**A**) Box-and-whisker plots with overlaid individual data points compare the total number of symptoms between the post-COVID-19 group (median 4, IQR 2–7, *n* = 201) and the control group (median 2, IQR 1–5, *n* = 201). Groups were compared using the two-sided Mann–Whitney U test (U = 26,564, *p* < 0.0001). (**B**) Box-and-whisker plots with overlaid individual data points compare the same outcome across the post-COVID-19 group (median 4, IQR 2–7, *n* = 201), anti-N-negative controls (median 2, IQR 1–4, *n* = 73), and anti-N-positive controls (median 3, IQR 1–6, *n* = 64). Groups were compared using the Kruskal–Wallis test (H = 20.43, *p* < 0.0001). Boxes represent the interquartile range, center lines indicate medians, whiskers extend to 1.5 × IQR, and points represent individual observations.

To compare symptom burden between individuals after COVID-19 and a population-based control group, we constructed a 23-item composite symptom count reported in the last 6 months (range 0–23). The component symptom domains and their item definitions are provided in Supplementary Table S3. The study and control cohorts were matched on a two-month calendar window of assessment to minimize the impact of seasonal influences. The distribution of counts was right-skewed across groups, with higher values in the post-COVID-19 cohort (median 4 symptoms; *n* = 201) than in the control (all) cohort (median 2; *n* = 201). Within controls, anti-N serology revealed a graded pattern: anti-N-negative participants reported a median of 2 symptoms (*n* = 73), whereas anti-N-positive participants reported 3 (*n* = 64), falling between anti-N negative controls and the post-COVID-19 group. Results are shown in Figure 3. Anti-N-positive and anti-N-negative controls did not differ significantly in available baseline characteristics, including age, sex, BMI, smoking status, or two-month visit window; this comparison is provided in Supplementary Table S6.

Using the a priori domain classification described in Section 2 and in Supplementary Table S3, the 23 questionnaire items were grouped into 7 symptom domains (fatigue; inflammatory; respiratory; neurocognitive; cardiovascular; allergic; dermatologic) to reduce multiplicity and group clinically related symptoms. As shown in Table 3, domain-level prevalence (≥1 symptom) was consistently higher in the post-COVID-19 group than in controls after adjustment for age, sex, and BMI. The largest effects were observed for the dermatologic domain, followed by fatigue, allergic, neurocognitive, and cardiovascular; the respiratory domain showed a similar but weaker pattern, and the inflammatory domain differed only modestly. Available denominators for symptom-domain analyses were 158 controls and 175 post-COVID-19 participants for most domains; for the inflammatory domain, data were available for 158 controls and 182 post-COVID-19 participants.

Item-level modeling clarified which specific symptoms drove these domain-level signals: hair loss in the dermatologic domain, memory impairment in neurocognitive, fatigue in fatigue, fever in inflammatory, and palpitations in cardiovascular. Full per-item results (top driver per domain) are provided in Supplementary Table S4, and domain-burden distributions (proportion scores with Wilcoxon tests) are reported in Table S5.

Within the post-COVID-19 cohort, determinants of multisystem burden—operationalized as the number of positive domains (0–7)—were evaluated using negative binomial regression (Table 4). Female sex was independently associated with a higher symptom burden, whereas age, BMI, hospitalization in the acute phase, and the composite severe acute biomarker definition (CRP ≥ 100 mg/L, IL-6 ≥ 80 pg/mL, D-dimer ≥ 1000 ng/mL) were not associated with the count of affected domains at 6–8 months. In an additional exploratory sensitivity analysis adjusted for smoking status, smoking was not significantly associated with the number of affected domains, and the overall pattern of associations remained broadly similar. Given the reduced complete-case sample and the small number of smokers in the post-COVID-19 group, this analysis was considered exploratory.

## 4. Discussion

Long COVID (post-COVID-19 condition) is a syndrome characterized by persistent or recurrent symptoms following SARS-CoV-2 infection, typically occurring ~3 months after the onset of the disease, lasting at least 2 months, and not explained by another diagnosis [11,12]. For the purposes of the present study, we used a time-based framework distinguishing the acute phase, the early post-acute assessment at approximately 4 weeks, and the late post-acute assessment at 6–8 months, which facilitates interpretation of symptom trajectories. Long COVID is a multisystem syndrome with varying dynamics and duration, described in the literature as encompassing > 200 symptoms and affecting multiple systems. Reviews and cohort studies most commonly report clusters of fatigue and neurocognitive symptoms as well as features of cardiovascular dysautonomia [26,27,28,29]. Meta-analyses and reviews indicate a wide range of prevalence (depending on the definition, population, and time point, among other factors), but confirm the persistence of symptoms in a significant proportion of recovered patients [30,31,32]. Proposed mechanisms include persistent immune activation, microcirculation disorders, dysautonomia, viral reservoir and the consequences of organ damage after the acute phase [27,33]. Clusters of symptoms are often described (e.g., fatigue–systemic, neurocognitive, respiratory–inflammatory, cardiac–autonomic), and some symptoms have different recovery trajectories—e.g., the sense of smell returns to normal in most people within 6 months, but objective tests show that dysfunction persists in subgroups [6,26]. Significant risk factors include female gender and previous symptom/comorbidity profile; meta-analysis results confirm a higher risk in women [34,35]. Beyond the health dimension, the social and economic significance of long COVID is also growing, including productivity costs and labor market impacts [36]. Methodologically, researchers use different approaches to assess long COVID: (I) longitudinal projects tracking symptom trajectories, (II) clustering/phenotyping to capture heterogeneity, and (III) comparisons with population controls (including ‘never-infected’ or test-negative) to attribute symptoms to past infection [6,32,37,38,39,40]. Some of these studies also control for time/period (e.g., analyzing by variant periods), which helps to limit seasonal confounding of common ailments [37,38,39].

In contrast to previous analyses of long COVID, which typically employ symptom clustering or comparisons with population groups matched by basic characteristics such as age, sex, or month of testing, this study uniquely matches participants by seasonality of visit (two-month windows). However, although season matching likely reduced background seasonal variation in common respiratory symptoms, it could not fully account for individual-level concurrent respiratory infections or SARS-CoV-2 reinfections during follow-up. While it is possible that a history of COVID-19, particularly hospitalization, could influence patients to attribute symptoms occurring six months later to that event, the increased symptom burden observed in controls from the general population cohort, who were unaware of their infection but demonstrated elevated anti-N antibodies, suggests that these findings are unlikely to be solely due to psychosomatic attribution.

Analysis of the trajectory of COVID-19 symptoms in our cohort showed that the frequency of coughing and shortness of breath decreased rapidly between the acute phase and ~4 weeks and remained low for 6–8 months, while the return of smell/taste function was slower and incomplete in some of the subjects; greater fatigue also persisted, with little change in anxiety symptoms (Figure 2). This separation of lines—early normalization of the respiratory component with prolonged systemic and neurocognitive symptoms—is consistent with the course of post-COVID-19 condition (PCC) in longitudinal studies, which describe diverse trajectories of symptom domains up to 24 months after infection [41]. In the Scottish Long-COVID in Scotland Study (Long-CISS) [37] cohort (with a ‘never-infected’ comparison group), persistent health limitations and symptoms were recorded at 6/12/18 months, confirming that in some patients, improvement is incomplete despite the passage of time, consistent with our observations of sustained respiratory improvement with greater persistence of generalized symptoms [19,42]. However, in comparison with the previously mentioned study, respiratory symptoms normalized earlier in our analyses; this discrepancy can probably be attributed to differences in clinical profiles and outcome assessment (our study included acute clinical markers, while Long-CISS is based on repeated Patient-Reported Outcome Measures (PROMs) and population-level observations). Furthermore, in the Dutch Lifelines cohort [39], after adjusting for pre-infection status and symptom dynamics in the uninfected population, a set of symptoms typical of PCC (including fatigue, weakness, memory impairment) persisted, which corresponds to the slower resolution of fatigue observed in our study. The similarity also applies to the sensory profile: in their review, Dias et al. [26] emphasized that, at the self-report level, the percentage of people declaring a return of smell within 6 months reaches ~95%, but psychophysical tests reveal a significant percentage of persistent dysfunction—our course of ‘monotonous improvement with residual deficit in a minority’ in 6–8 months is consistent with this [43]; similar conclusions were reported by Tervo et al. and other studies with objective measurements [44,45]. The divergence we observe between dyspnea and fatigue—namely, a more rapid decline in dyspnea with slower resolution of fatigue—aligns with 12–24-month studies showing that respiratory recovery precedes reductions in systemic symptoms, with the lowest health-related quality-of-life indices typically occurring at 6–12 months [41,46]. In the area of mental health, reports of stable trajectory classes (often with persistently higher levels of anxiety in subgroups) are consistent with the low variability in anxiety severity in our cohort [47].

At 6–8 months, our cohort showed a higher prevalence of cardiovascular/autonomic symptoms than season-matched controls (adjusted OR 1.75, 95% CI 1.15–2.66; Table 2), with palpitations and other orthostatic-type complaints among the leading item-level contributors (Supplement Table S4). Although we did not perform formal autonomic testing, this pattern is consistent with reports of dysautonomia, including Postural Orthostatic Tachycardia Syndrome (POTS)-like presentations, in post-COVID-19 condition and should be regarded as hypothesis-generating rather than confirmatory in our cohort [26,48]. Clinically, our findings support a pragmatic approach: outpatient follow-up should use structured multisystem symptom screening rather than focus only on respiratory complaints. In stable patients without alarm features, clinicians may de-emphasize intensive pulmonary investigations and prioritize functional rehabilitation; fatigue management; and assessment of neurocognitive, cardiovascular/autonomic, olfactory, and dermatologic complaints. When olfactory deficits persist, monitor and treat them using objective assessments; and in patients with chronic fatigue, palpitations, or orthostatic intolerance, consider targeted evaluation of autonomic function [26,45,48,49]. Importantly, while most studies control for demographics, our research introduces seasonal adjustment of visit timing (two-month windows), which reduces the risk of seasonal bias in typical complaints and strengthens the attribution of intergroup differences. Importantly, we did not find an association between a severe course of the acute phase of COVID-19 and persistence of symptoms. This may suggest different mechanisms underlying the acute phase course of the disease and PCC.

At six to eight months, we observed a higher overall symptom burden in the post-COVID-19 group versus season-matched controls, with an intermediate burden in anti-N-positive controls (Figure 3). The overall symptom burden in our post-COVID-19 group (median four symptoms) exceeded that of season-matched controls (median two), consistent with the Swiss population cohort [41] reporting an adjusted risk difference of 17.0% for symptoms at 6 months in infected vs. uninfected participants; the largest excesses there involved altered smell/taste, dyspnea, reduced concentration, memory problems, and post-exertional malaise. By contrast, our respiratory domain did not differ significantly between groups at 6–8 months. This finding likely reflects a combination of true respiratory recovery and methodological factors rather than the complete absence of residual respiratory complaints. In the longitudinal hospitalized subgroup, cough and dyspnea declined rapidly after the acute phase and remained low at 6–8 months. This interpretation is also consistent with our previous chest CT and pulmonary function study six months after COVID-19, in which most CT abnormalities had resolved, and pulmonary function test results in convalescents were comparable to those observed in a representative population cohort [50]. More broadly, several longitudinal studies have shown objective pulmonary improvement within 3–6 months, which may attenuate between-group contrasts by the half-year mark [51,52]. Additionally, season matching likely reduced false differences in cough/upper-airway symptoms, which are strongly seasonal in the general population [53]. Moreover, because respiratory-domain symptoms were relatively infrequent at follow-up and were based on patient report, small residual differences may have been difficult to detect. Finally, as SARS-CoV-2 has evolved, its symptom profile has converged toward other respiratory viruses, further diminishing COVID-specific respiratory signals against a seasonal background [54].

According to our analyses, hair loss emerged as one of the most frequently reported late symptoms in our cohort and was a principal contributor to the dermatologic domain (Table 2; Supplement Table S4). Population evidence supports this signal. In a nationwide cohort study, Kim et al. [55] reported a significant post-COVID-19 increase in both incidence and prevalence of Alopecia Areata (AA), and—importantly for our findings—an elevated risk of Telogen Effluvium (TE) in infected versus matched uninfected controls (adjusted HR 6.40; 95% CI 4.92–8.33), after adjustment for multiple confounders; proposed mechanisms for COVID-19-associated hair loss include molecular mimicry, cytokine-profile shifts, and bystander activation of autoimmunity. Mechanistically, the prominence of hair loss reported by study participants at our 6–8-month follow-up might be compatible with the natural history of post-stress TE, which typically begins ~2–3 months after the trigger (fever/systemic inflammation, metabolic and psychosocial stress) and persists beyond 6 months in a subset of features repeatedly described in post-COVID-19 cohorts [56,57]. Complementary observational and review data further document TE as a common post-COVID-19 manifestation, including trichoscopy-confirmed series and systematic reviews [58,59]. We did not perform formal dermatologic phenotyping (e.g., trichoscopy) to distinguish AA versus TE, which limits mechanistic attribution; however, the high frequency of reported hair shedding in our post-COVID-19 group, together with external evidence, underscores the need for targeted dermatologic evaluation and longitudinal follow-up in this population.

In addition, serological testing in both cohorts enabled identification of control individuals without a recorded history of COVID-19 who nevertheless tested antibody-positive and exhibited an intermediate symptom burden between seronegative controls and clinically recognized post-COVID-19 cases. This may suggest that unrecognized (often asymptomatic or with few symptoms) infections may still cause subsequent ailments. This serological identification is robust to the principal sources of exposure misclassification: anti-nucleocapsid antibodies are induced only by natural SARS-CoV-2 infection and not by the spike-targeted vaccines administered in Poland during the study period, and the Elecsys assay used has a manufacturer-reported specificity above 99% [60], rendering analytical false positives negligible at the cohort level. There is extensive evidence that the long-term sequelae of COVID-19 scale with the acute severity of SARS-CoV-2 infection—i.e., more severe acute disease is associated with higher risk and greater burden of post-COVID-19 condition (PCC) [21]. Other studies have identified the number of symptoms at onset as an independent risk factor for subsequent PCC. Because inflammatory and thrombotic markers, including CRP, IL-6, and D-dimer, are associated with acute COVID-19 severity [25], we assessed whether a biomarker-defined severe acute course was also related to later multisystem symptom burden. In our analyses, however, neither acute-phase severity nor the initial symptom count was significantly associated with outcomes at six to eight months [61]. This null finding is plausibly explained by our case mix and power: the cohort contained few individuals with severe acute illness and was composed predominantly of mild-to-moderate cases, constraining exposure variability and reducing the ability to detect graded associations. It may also indicate partial pathophysiological dissociation between the mechanisms captured by routine acute inflammatory/thrombotic biomarkers and those responsible for persistent post-COVID-19 symptoms, such as autonomic, neuroimmune, endothelial, or post-infectious recovery pathways. Therefore, our findings should not be interpreted as excluding a role of acute inflammation, but rather, as showing that these routinely available acute-phase biomarkers were not sufficient predictors of later multisystem symptom burden in this cohort. Ongoing work continues to refine PCC risk stratification and to elucidate mechanisms—including those relevant to previously unrecognized SARS-CoV-2 infection—which should ultimately inform earlier identification and more targeted post-infectious evaluation in future practice.

Nevertheless, among the prognostic factors evaluated, female sex was independently associated with a greater burden of multisystem symptoms at 6–8 months, whereas hospitalization and the biomarker-defined severe acute course were not. This is consistent with the observations of Fernández-de-las-Peñas et al. [35], who reported a higher risk of post-COVID-19 symptoms in women despite no overall gender differences in acute COVID-19 symptoms at the time of hospital admission. Similarly, recent data from the NIH RECOVER-Adult cohort showed that female sex was associated with a higher risk of long COVID despite the generally higher acute COVID-19 severity and mortality observed in men [62]. In summary, these findings suggest that female sex is a more likely risk factor for persistent symptoms after acute illness than for the initial clinical presentation [63]. Several non-mutually-exclusive mechanisms may underlie this pattern: sex-related biological differences (e.g., differences in angiotensin converting enzyme 2 (ACE2) and transmembrane protease, serine 2 (TMPRSS2) expression) and immune response profiles (including relatively lower IL-6 production after viral infection in women) may influence the course of recovery [64,65,66,67]. Methodological factors should also be considered, including self-reported symptom assessment and possible sex-related differences in symptom perception, reporting, healthcare seeking, or follow-up participation. In addition, our acute severity measures captured hospitalization status and selected inflammatory/thrombotic biomarkers but may not fully reflect other acute-phase features relevant to PCC, such as symptom intensity, viral load, immune phenotype, or tissue-specific injury. Although behavioral explanations, such as more frequent hand washing or lower exposure risk, have been proposed, they are unlikely to explain the observed excess prevalence of long-term symptoms [68]. Pandemic-related factors, including social isolation, psychological stress and reduced physical activity, may have disproportionately affected women and contributed to a higher burden of post-traumatic symptoms [69].

Although our conclusions are consistent, several issues require clarification in their interpretation. First, we relied on self-reported symptoms collected through a structured but non-validated interview, with attendant potential for recall and reporting bias; future studies should be supplemented with objective measurements (psychophysical smell tests, standardized cognitive tests, spirometry/DLCO). Second, we did not perform standardized tests of the autonomic nervous system. In future projects, tilt table testing and indicators of autonomic regulation (e.g., heart rate variability) should be considered. Third, the 6–8-month assessment point limits insight into longer-term dynamics; 12–36-month observations with repeated measurements are needed. Fourth, 80.1% of participants in the post-COVID-19 cohort had been hospitalized during the acute phase, which may limit the generalizability of our findings to non-hospitalized individuals with milder COVID-19. Although a recent meta-analysis indicates that overall long COVID prevalence is broadly comparable between hospitalized and non-hospitalized survivors at medium-term follow-up [70], the symptom profile differs partially: respiratory and chest symptoms tend to be more pronounced after hospitalization, whereas fatigue, neurocognitive, and dermatologic complaints occur at broadly comparable rates regardless of acute care setting. The dominant non-respiratory excess burden observed in our cohort is therefore likely to extend qualitatively to milder disease, although absolute prevalences in unselected outpatient populations would be expected to be lower than those reported here. At the same time, the proportion with biomarker-defined severe disease was relatively small, which may have limited our ability to detect associations between acute severity and later symptom burden. Fifth, although smoking status was collected in both cohorts, these data remained incomplete, and the number of smokers in the post-COVID-19 group was relatively small. For this reason, smoking status was not included in the primary multivariable models. Instead, smoking-adjusted analyses were performed as exploratory complete-case sensitivity analyses. In these exploratory models, smoking was not significantly associated with multisystem symptom burden; however, residual confounding related to smoking cannot be fully excluded. Sixth, although season-matched controls reduced confounding related to seasonal variation in respiratory and systemic symptoms, we did not systematically collect data on other concurrent respiratory infections or SARS-CoV-2 reinfections during follow-up. Because the post-COVID-19 study protocol and questionnaires were developed early in the pandemic, these exposures were not explicitly captured. Their potential contribution to the reported symptom burden, particularly in respiratory domains, therefore, cannot be excluded. Seventh, viral variants, vaccination status, and acute-phase treatment were not systematically incorporated into the analyses. These factors may have influenced acute disease severity, biomarker levels, symptom profiles, and recovery trajectories; therefore, residual confounding by pandemic period and treatment context cannot be excluded. Despite these limitations, season-matched assessment and serological verification of control participants remain important strengths that support the reliability of our findings.

## 5. Conclusions

Across a seasonally adjusted control study, we demonstrated that 6–8 months after COVID-19, there is a greater multisystemic burden of symptoms than in population controls, with a predominance of fatigue, hair loss, neurocognitive and cardiac autonomic complaints. Trajectory analysis among hospitalized patients indicates a predominance of respiratory symptoms in the acute phase, a marked decline after discharge, and a partial recurrence approximately 6–8 months after infection. Fatigue/weakness temporarily resolves after discharge but recurs and remains the main chronic complication. Individuals with an asymptomatic acute phase showed an intermediate burden of late symptoms compared with the post-COVID-19 group and seronegative controls. Female gender was independently associated with greater severity of long COVID symptoms. These findings support the establishment of structured, gender-specific pathways for further rehabilitation programs that prioritize fatigue management and screening for autonomic dysfunction. Larger, longitudinal cohorts with biomarker and mechanism profiling are needed to elucidate causal pathways and test targeted interventions capable of preventing or reducing long-term morbidity.

## Figures and Tables

**Table 1:** Baseline characteristics of study population.

Variable	Control	Post-COVID-19	*p*-Value
Age, years	50.6 ± 14.1 (Mdn 52.0)	51.9 ± 12.8 (Mdn 52.0)	0.416
Female, *n* (%)	94 (46.8%)	94 (46.8%)	1.000
BMI, kg/m^2^	27.2 ± 5.4 (Mdn 26.6)	29.8 ± 6.1 (Mdn 29.3)	<0.001
Current smoking	53/158 (33.5%)	21/171 (12.3%)	<0.001
Visit period, *n* (%)			1.000
Janurary–February ^1^	9 (4.5%)	9 (4.5%)	
March–April ^2^	7 (3.5%)	7 (3.5%)	
May–June ^3^	37 (18.4%)	37 (18.4%)	
July–August ^4^	71 (35.3%)	71 (35.3%)	
September–October ^5^	35 (17.4%)	35 (17.4%)	
November–December ^6^	42 (20.9%)	42 (20.9%)	
Anti-N serostatus	Positive: 64 (46.7%); Negative: 73 (53.3%); Missing: 64	Positive: 182 (96.8%); Negative: 6 (3.2%); Missing: 13	<0.001

Variables are shown as mean ± SD (median); categorical variables are *n* (%); percentages use available cases. Visit period reflects the bimonthly calendar window used for 1:1 matching: ^1^ = January–February, ^2^ = March–April, ^3^ = May–June, ^4^ = July–August, ^5^ = September–October, ^6^ = November–December. Anti-N serostatus: anti-nucleocapsid SARS-CoV-2 antibodies (positive/negative); missing = no result. *p*-Value compares post-COVID-19 vs. control; Wilcoxon rank-sum for continuous and χ^2^ (or Fisher’s exact when appropriate) for categorical; two-sided. Abbreviations: BMI, body mass index; SD, standard deviation; Mdn, median.

**Table 2:** Acute-phase characteristics among participants in the post-COVID-19 group.

Variable	Post-COVID-19
Hospitalized group, *n* (%)	161 (80.1%)
Severe COVID-19	26/161 (16.1%)
CRP mg/L	55.1 [19.7–118.5] (*n* = 157)
IL-6 pg/mL	43.9 [20.6–102.5] (*n* = 140)
D-dimer ng/mL	1007.5 [563.5–1640.5] (*n* = 156)

Data are *n* (%) or median [IQR]. Biomarkers represent peak in-hospital values. A composite threshold defined severe COVID-19: CRP ≥ 100 mg/L, IL-6 ≥ 80 pg/mL, and D-dimer ≥ 1000 ng/mL [25].

**Table 3:** Symptom domains at 6–8-month follow-up: post-COVID-19 vs. control.

Domain	Control *n*/*N* (%)	Post-COVID-19 *n*/*N* (%)	aOR (95% CI)	*p*-Value (aOR)
Allergic	58/158 (36.7%)	91/175 (52.0%)	2.22 (1.44–3.40)	<0.001
Dermatologic	32/158 (20.3%)	76/175 (43.4%)	4.15 (2.42–7.11)	<0.001
Fatigue	47/158 (29.7%)	89/175 (50.9%)	2.64 (1.67–4.16)	<0.001
Neurocognitive	56/158 (35.4%)	94/175 (53.7%)	2.16 (1.40–3.33)	0.001
Cardiovascular	81/158 (51.3%)	114/175 (65.1%)	1.75 (1.15–2.66)	0.010
Inflammatory	73/158 (46.2%)	99/182 (54.4%)	1.55 (1.03–2.34)	0.037
Respiratory	12/158 (7.6%)	27/175 (15.4%)	1.90 (0.90–3.99)	0.090

For each domain, prevalence of ≥1 symptom is shown as *n*/*N* (%), where N denotes participants with available data for the respective domain. The primary inference is from multivariable logistic regression using complete cases for the variables included in each model. Models were adjusted for age, sex, and BMI; estimates are reported as aORs with 95% CIs and two-sided Wald *p*-values.

**Table 4:** Predictors of multisystem symptom burden at 6–8 months in the post-COVID-19 cohort.

Predictor	IRR (95% CI)	*p*-Value
Age (per 10 years)	1.02 (0.90–1.16)	0.743
BMI (per 5 kg/m^2^)	1.09 (0.95–1.24)	0.236
Female (vs. male)	1.65 (1.17–2.31)	0.004
Hospitalized (acute)	1.02 (0.67–1.56)	0.915
Severe acute course (CRP ≥ 100, IL-6 ≥ 80, D-dimer ≥ 1000)	1.05 (0.64–1.75)	0.838

Outcome: number of positive symptom domains at 6–8 months (0–7; domain positive = ≥ 1 “Yes”). Model: multivariable negative binomial; results shown as IRR (95% CI) with two-sided Wald *p*. Covariates: age (per 10 years), BMI (per 5 kg/m^2^), female sex (vs. male), acute-phase hospitalization (yes/no), and severe biomarker profile (CRP ≥ 100 mg/L, IL-6 ≥ 80 pg/mL, D-dimer ≥ 1000 ng/mL; all met = 1).

## Data Availability

The original contributions presented in this study are included in the article. Further inquiries can be directed to the corresponding author.

## Supplementary Materials

The following supporting information can be downloaded at: https://www.mdpi.com/article/10.3390/jcm15103707/s1, Table S1. Inclusion, matching, and exclusion criteria for the post-COVID-19 and control cohorts; Table S2: Paired within-person changes and age/sex-adjusted time effects for five symptoms across study visits (McNemar OR and GEE aOR, 95% CI); Table S3: Symptom items used to define the symptom domains at the 6–8-month follow-up; Table S4: Item-level adjusted associations for symptom items within domains at the 6–8-month follow-up; Table S5: Domain-burden distributions at the 6–8-month follow-up: proportion of positive items among answered items, by group; Table S6. Baseline characteristics of anti-N-negative and anti-N-positive controls.

## Abbreviations

The following abbreviations are used in this manuscript:

ACE2	Angiotensin-converting enzyme 2
anti-N	Anti-nucleocapsid (SARS-CoV-2) antibodies
aOR	Adjusted odds ratio
ARDS	Acute respiratory distress syndrome
BMI	Body mass index
CI	Confidence interval
COVID-19	Coronavirus disease 2019
CRP	C reactive protein
ECLIA	Electrochemiluminescence immunoassay
FDR	False discovery rate
GEE	Generalized estimating equations
HR	Hazard ratio
IL-6	Interleukin-6
IQR	Interquartile range
IRR	Incidence rate ratio
IgG	Immunoglobulin G
IgM	Immunoglobulin M
Mdn	Median
NICE	National Institute for Health and Care Excellence
PCC	Post-COVID-19 condition
POTS	Postural orthostatic tachycardia syndrome
PROMs	Patient-Reported Outcome Measures
RT-PCR	Reverse transcriptase chain reaction
SARS-CoV-2	Severe acute respiratory syndrome coronavirus 2
SD	Standard deviation
TMPRSS2	Transmembrane protease, serine 2
