# Supplementary material for: Symptom Trajectories and Long-Term Sequelae of COVID-19: A Matched Case–Control Study with Population-Based Controls
Source: J Clin Med. 2026 May 12;15(10):3707. doi: 10.3390/jcm15103707 (PMC13207038; doi:10.3390/jcm15103707)
Supplement: Supplementary file 1 [file jcm-15-03707-s001.zip › jcm-4281777-supplementary.pdf]

Supplementary Materials

Table S1. Inclusion, matching, and exclusion criteria for the post-COVID-19 and control cohorts.

Criterion	Post-COVID-19 group	Control group
**Inclusion criteria**		
**Age**	Adults ≥18 years	Adults ≥18 years
**Exposure / source**	RT-PCR-confirmed SARS-CoV-2 infection (nasopharyngeal swab)	Participation in the population-based Bialystok PLUS study; no self-reported history of COVID-19
**Acute infection period**	April 2020 – February 2022	Not applicable
**Examination period (this study)**	November 2020 – September 2022 (6–8 months post-acute)	January 2020 – December 2022
**Follow-up requirement**	Attendance at the 6–8-month follow-up visit	Attendance at a Bialystok PLUS visit within the matched calendar window
**Matching criteria**		
**Variables**	—	Age, sex, and two-month calendar window of examination
**Exclusion criteria (applied identically to both groups)**		
**History of any of:**	• Myocardial infarction<br>• Stroke<br>• Psoriasis<br>• Vitiligo<br>• Type 1 diabetes mellitus<br>• Crohn's disease<br>• Allergic disease<br>• Chronic obstructive pulmonary disease<br>• HIV infection<br>• Hepatitis B<br>• Hepatitis C<br>• Thyroid dysfunction<br>• Rheumatoid arthritis<br>• Systemic lupus erythematosus<br>• Any cancer	

All criteria were applied prior to data analysis. The two-month matching window was selected as the narrowest temporal criterion that preserved 1:1 pair retention within the available Bialystok PLUS control pool whilst limiting confounding by seasonal respiratory and allergic symptoms (see Section 2.1). Exclusion criteria were applied identically to participants in both groups.

Table S2. Paired within-person changes and age/sex-adjusted time effects for five symptoms across study visits (McNemar OR and GEE aOR, 95% CI).

Symptom	Time contrast	Pairs (n)	Paired OR (McNemar) [95% CI]	p (Holm)	Adjusted OR (GEE, age+sex) [95% CI]
Cough	4 weeks vs Hospitalisation	106	78.00 (10.85–560.73)	<0.001	0.03 (0.01–0.05)
Cough	6-8 months vs Hospitalisation	76	10.33 (3.16–33.80)	<0.001	0.14 (0.08–0.25)
Dyspnoea	4 weeks vs Hospitalisation	105	4.64 (2.42–8.90)	<0.001	0.26 (0.15–0.44)
Dyspnoea	6-8 months vs Hospitalisation	75	3.38 (1.53–7.43)	0.015	0.26 (0.15–0.44)
Anxiety symptoms	4 weeks vs Hospitalisation	97	0.50 (0.05–5.51)	1	1.01 (0.36–2.88)
Anxiety symptoms	6-8 months vs Hospitalisation	64	0.09 (0.01–0.70)	0.044	7.92 (2.24–27.97)
Fatigue	4 weeks vs Hospitalisation	107	7.86 (3.58–17.25)	<0.001	0.16 (0.09–0.29)
Fatigue	6-8 months vs Hospitalisation	75	0.38 (0.14–1.08)	0.481	1.54 (0.72–3.29)
Smell/Taste disorder	4 weeks vs Hospitalisation	105	9.00 (2.09–38.79)	0.004	0.18 (0.06–0.52)
Smell/Taste disorder	6-8 months vs Hospitalisation	75	0.75 (0.26–2.16)	1	1.05 (0.55–1.98)

Table S3. Symptom items used to define the symptom domains at the 6-8-month follow-up.

Domain	Component symptom items
**Fatigue**	1. Fatigue
**Inflammatory**	2. Lymphadenopathy (enlarged lymph nodes)<br>3. Fever<br>4. Night sweats<br>5. Hyperhidrosis (increased sweating)
**Respiratory**	6. Bendopnea (dyspnea on bending forward)<br>7. Positional dyspnea (dyspnea when lying on the left side)
**Neurocognitive**	8. Dizziness/balance disturbances<br>9. Visuomotor incoordination<br>10. Memory impairment
**Cardiovascular**	11. Pallor and coldness of hands and feet<br>12. Increased blood pressure<br>13. Decreased blood pressure<br>14. Blanching or cyanosis of the fingers<br>15. Dependent peripheral edema (increasing leg or ankle oedema during the day)<br>16. Palpitations
**Allergic**	17. Sneezing<br>18. Nasal pruritus<br>19. Rhinorrhea (excessive nasal discharge)<br>20. Nasal obstruction<br>21. Ocular burning, lacrimation, eyelid edema
**Dermatologic**	22. Eczema/skin rash<br>23. Hair loss

Table S4. Item-level adjusted associations for symptom items within domains at the 6-8-month follow-up.

Domain	Item	Control n(%)	Post-COVID n(%)	p (unadj)	aOR (95% CI)	p (adj)	q (FDR, within domain)
Fatigue	Fatigue	47 (29.7%)	89 (50.9%)	0.000	2.41 (1.50–3.87)	<0.001	0.000
Inflammatory	Fever	27 (17.1%)	60 (34.3%)	0.001	2.57 (1.50–4.41)	0.001	0.002
Inflammatory	Night sweats	40 (25.3%)	56 (32.0%)	0.221	1.29 (0.79–2.11)	0.307	0.425
Inflammatory	Lymphadenopathy	13 (8.2%)	9 (4.9%)	0.314	0.63 (0.25–1.57)	0.318	0.425
Inflammatory	Hyperhidrosis	39 (24.7%)	42 (24.0%)	0.986	0.87 (0.52–1.45)	0.589	0.589
Respiratory	Positional dyspnea (dyspnea when lying on the left side)	2 (1.3%)	11 (6.3%)	0.022	4.63 (1.00–21.50)	0.051	0.101
Respiratory	Bendopnea	11 (7.0%)	24 (13.7%)	0.068	1.60 (0.73–3.53)	0.243	0.243
Neurocognitive	Memory impairment	29 (18.4%)	77 (44.0%)	<0.001	3.64 (2.16–6.14)	<0.001	<0.001
Neurocognitive	Visuomotor incoordination	2 (1.3%)	10 (5.7%)	0.038	3.93 (0.83–18.63)	0.085	0.127
Neurocognitive	Dizzines/ balance disturbances	40 (25.3%)	52 (29.7%)	0.439	1.10 (0.67–1.83)	0.697	0.697
Cardiovascular	Palpitations	25 (15.8%)	55 (31.4%)	0.001	2.33 (1.34–4.03)	0.003	0.016
Cardiovascular	Decreased blood pressure	6 (3.8%)	15 (8.6%)	0.118	2.51 (0.93–6.78)	0.069	0.208
Cardiovascular	Dependent peripheral edema	35 (22.2%)	60 (34.3%)	0.020	1.48 (0.86–2.52)	0.155	0.310
Cardiovascular	Increased blood pressure	25 (15.8%)	41 (23.4%)	0.109	1.44 (0.80–2.58)	0.222	0.333
Cardiovascular	Pallor and coldness of hands and feet	25 (15.8%)	22 (12.6%)	0.488	0.79 (0.42–1.51)	0.484	0.580
Cardiovascular	Blanching or cyanosis of the fingers	5 (3.2%)	6 (3.4%)	1.000	1.10 (0.32–3.80)	0.876	0.876
Allergic	Nasal pruritus	11 (7.0%)	23 (13.1%)	0.093	2.03 (0.93–4.41)	0.074	0.281
Allergic	Nasal obstruction	32 (20.3%)	45 (25.7%)	0.294	1.55 (0.90–2.66)	0.112	0.281
Allergic	Ocular burning, lacrimation, eyelid edema	34 (21.5%)	44 (25.1%)	0.516	1.29 (0.76–2.18)	0.343	0.559
Allergic	Sneezing	26 (16.5%)	33 (18.9%)	0.668	1.19 (0.67–2.13)	0.554	0.559
Allergic	Rhinorrhea	23 (14.6%)	27 (15.4%)	0.945	1.20 (0.65–2.24)	0.559	0.559
Dermatologic	Hair loss	22 (13.9%)	67 (38.3%)	<0.001	4.77 (2.61–8.72)	<0.001	<0.001
Dermatologic	Eczema/skin rash	13 (8.2%)	15 (8.6%)	1.000	1.09 (0.48–2.45)	0.838	0.838

Table S5. Domain-burden distributions at the 6-8-month follow-up: proportion of positive items among answered items, by group.

Domain	Control (score)	Post-COVID (score)	p (Wilcoxon)	N (Control / Post-COVID)
Allergic	0.00 [0.00–0.20]	0.20 [0.00–0.20]	0.034	158 / 175
Dermatologic	0.00 [0.00–0.00]	0.00 [0.00–0.50]	<0.001	158 / 175
Fatigue	0.00 [0.00–1.00]	1.00 [0.00–1.00]	<0.001	158 / 175
Neurocognitive	0.00 [0.00–0.33]	0.33 [0.00–0.33]	<0.001	158 / 175
Cardiovascular	0.17 [0.00–0.17]	0.17 [0.00–0.33]	0.001	158 / 175
Inflammatory	0.00 [0.00–0.25]	0.25 [0.00–0.50]	0.116	158 / 182
Respiratory	0.00 [0.00–0.00]	0.00 [0.00–0.00]	0.022	158 / 175

Table S6. Baseline characteristics of anti-N-negative and anti-N-positive controls.

Variable	Anti-N-negative controls	Anti-N-positive controls	p-Value
**n**	73	64	—
**Age, years**	52.9 ± 14.2 (Mdn 57.0)	49.7 ± 14.1 (Mdn 47.0)	0.165
**Female, n (%)**	33 (45.2%)	30 (46.9%)	0.845
**BMI, kg/m²**	27.8 ± 5.9 (Mdn 27.3)	27.1 ± 5.3 (Mdn 26.7)	0.511
**Current smoking, n/N (%)**	18/64 (28.1%)	18/51 (35.3%)	0.410
	**Visit period, n (%)**		
**Jan–Feb**	3 (4.1%)	1 (1.6%)	
**Mar–Apr**	3 (4.1%)	3 (4.7%)	
**May–Jun**	19 (26.0%)	18 (28.1%)	0.953
**Jul–Aug**	22 (30.1%)	17 (26.6%)	
**Sep–Oct**	10 (13.7%)	10 (15.6%)	
**Nov–Dec**	16 (21.9%)	15 (23.4%)	
